# *catena*-Poly[[(triaqua­cadmium)-μ-1,4-phenyl­enediacetato-κ^4^ *O*,*O*′:*O*′′,*O*′′′] dihydrate]

**DOI:** 10.1107/S1600536811047842

**Published:** 2011-11-16

**Authors:** Jacob W. Uebler, Robert L. LaDuca

**Affiliations:** a Lyman Briggs College, Department of Chemistry, Michigan State University, East Lansing, MI 48825 USA

## Abstract

In the title compound, {[Cd(C_10_H_8_O_4_)(H_2_O)_3_]·2H_2_O}_*n*_, penta­gonal–bipyramidally coordinated Cd^II^ ions on a twofold rotation axis are linked by tethering 1,4-phenyl­enediacetate (1,4-phda) ligands into [Cd(1,4-phda)(H_2_O)_3_]_*n*_ coordination polymer chains. The chain motifs are oriented parallel to the *c*-axis direction. Individual chains are connected into a supra­molecular network *via* O—H⋯O hydrogen bonding involving the aqua ligands.

## Related literature

For other cadmium coordination polymers containing 1,4-phda ligands, see: Wang & LaDuca (2010 ▶); Farnum *et al.* (2011 ▶).
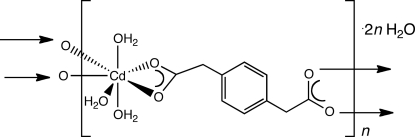


## Experimental

### 

#### Crystal data

[Cd(C_10_H_8_O_4_)(H_2_O)_3_]·2H_2_O
*M* *_r_* = 394.64
Monoclinic, 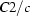

*a* = 7.6878 (7) Å
*b* = 8.2295 (8) Å
*c* = 22.735 (2) Å
β = 95.752 (1)°
*V* = 1431.1 (2) Å^3^
*Z* = 4
Mo *K*α radiation
μ = 1.57 mm^−1^
*T* = 173 K
0.34 × 0.32 × 0.29 mm

#### Data collection

Bruker APEXII CCD diffractometer
Absorption correction: multi-scan (*SADABS*; Sheldrick, 1996 ▶) *T*_min_ = 0.621, *T*_max_ = 0.659
11122 measured reflections
1307 independent reflections
1296 reflections with *I* > 2σ(*I*)
*R*_int_ = 0.025

#### Refinement

*R*[*F* ^2^ > 2σ(*F* ^2^)] = 0.013
*wR*(*F* ^2^) = 0.031
*S* = 1.19
1307 reflections
107 parameters
9 restraints
H atoms treated by a mixture of independent and constrained refinement
Δρ_max_ = 0.27 e Å^−3^
Δρ_min_ = −0.20 e Å^−3^

### 

Data collection: *APEX2* (Bruker, 2006 ▶); cell refinement: *SAINT* (Bruker, 2006 ▶); data reduction: *SAINT*; program(s) used to solve structure: *SHELXS97* (Sheldrick, 2008 ▶); program(s) used to refine structure: *SHELXL97* (Sheldrick, 2008 ▶); molecular graphics: *Crystal Maker* (Palmer, 2007 ▶); software used to prepare material for publication: *SHELXL97*.

## Supplementary Material

Crystal structure: contains datablock(s) I, global. DOI: 10.1107/S1600536811047842/ds2153sup1.cif

Structure factors: contains datablock(s) I. DOI: 10.1107/S1600536811047842/ds2153Isup2.hkl

Additional supplementary materials:  crystallographic information; 3D view; checkCIF report

## Figures and Tables

**Table 1:** Hydrogen-bond geometry (Å, °)

*D*—H⋯*A*	*D*—H	H⋯*A*	*D*⋯*A*	*D*—H⋯*A*
O1—H1⋯O4^i^	0.84 (1)	1.96 (1)	2.8029 (15)	178 (2)
O1*W*—H1*WA*⋯O2	0.84 (2)	1.86 (2)	2.6934 (17)	172 (2)
O4—H4*A*⋯O1*W*^ii^	0.83 (1)	1.89 (2)	2.7004 (17)	167 (2)
O4—H4*B*⋯O3^i^	0.84 (1)	1.84 (2)	2.6706 (16)	175 (2)

Symmetry codes: (i) 
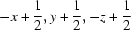
; (ii) 
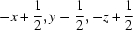
.

**Fig. 1.**
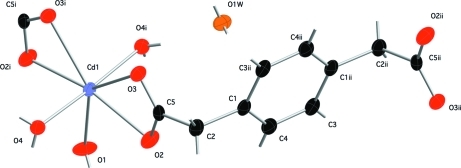
The coordination environment of the title compound, showing 50% probability ellipsoids and partial atom numbering scheme. Hydrogen atom positions are shown as grey sticks. Color codes: violet Cd, red bound O, orange unligated O, black C. Symmetry codes: (i) -x, y, -z + 1/2; (ii) -x, -y, -z.

**Fig. 2.**
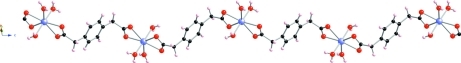
A single [Cd(1,4-phda)(H2O)3]n chain coordination polymer chain.

**Fig. 3.**
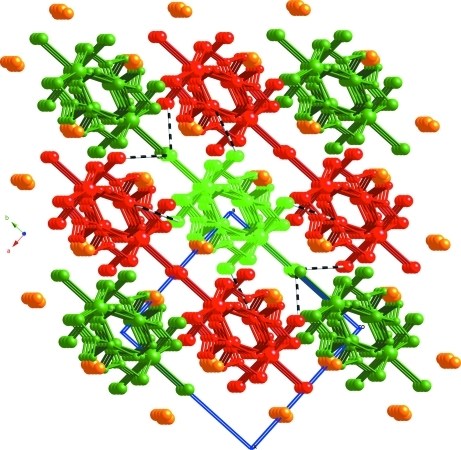
Supramolecular aggregation of [Cd(1,4-phda)(H2O)3]n chains. O—H···O hydrogen bonding is shown as dashed lines.

## supplementary crystallographic information

### Comment

Recently we have been investigating conformationally flexible phenylenediacetate ligands, especially 1,4-phenylenediacetate (1,4-phda), towards the construction of cadmium coordination polymers in tandem with dipodal nitrogen-base ligands (Wang & LaDuca, 2010; Farnum, *et al.*, 2011). The title compound was obtained upon an attempt to prepare a cadmium 1,4-phda coordination polymer incorporating 4,4'-trimethylenedipiperidine.

The asymmetric unit of the title compound contains a Cd^II^ ion and an aqua ligand on a 2-fold crystallographic rotation axis, an additional aqua ligand, half of a 1,4-phda ligand whose centroid lies on a crystallographic inversion center, and one water molecule of crystallization. The Cd^II^ ion is pentagonal bipyramidally coordinated, with its apical positions occupied by aqua ligands. Its equatorial positions contain a third aqua ligand and two chelating carboxylate groups from two 1,4-phda ligands (Fig. 1).

[Cd(H_2_O)_3_]^2+^ fragments are connected by exobidentate 1,4-phda ligands *via* a bis(chelating) binding mode, generating one-dimensional [Cd(1,4-phda)(H_2_O)_3_]_n_ coordination polymer chains (Fig. 2). Within the chain, the Cd···Cd contact distances measure 11.889 (6) Å. The chain motifs are all oriented parallel to the *c* crystal direction. Each individual [Cd(1,4-phda)(H_2_O)_3_]_n_ chain is anchored to four others *via* O—H···O hydrogen bonding mechanisms between aqua ligands in neighboring chains, and between aqua ligands and ligated 1,4-phda carboxylate oxygen atoms. In this manner, the supramolecular crystal structure of the title compound is constructed (Fig. 3).Water molecules of crystallization are held between coordination polymer chains through additional O—H···O hydrogen bonding interactions.

### Experimental

All starting materials were obtained commercially. A mixture of cadmium nitrate tetrahydrate (88 mg, 0.29 mmol), 1,4-phenylenediacetic acid (52 mg, 0.27 mmol), 4,4'-trimethylenedipiperidine (58 mg, 0.28 mmol) and 10.0 g water (550 mmol) was placed into a 23 ml Teflon-lined Parr acid digestion bomb, which was then heated under autogenous pressure at 393 K for 48 h. Colourless blocks of the title compound (57 mg, 0.14 mmol, 53% yield) were isolated after washing with distilled water and acetone, and drying in air.

### Refinement

All H atoms bound to C atoms were placed in calculated positions, with C—H = 0.95 Å, and refined in riding mode with *U*_iso_ = 1.2*U*_eq_(C). The H atoms bound to the aqua ligand O atom were found in a difference Fourier map, restrained with with O—H = 0.85 Å and refined with *U*_iso_ = 1.2*U*_eq_(O).

### Crystal data

**Table:**

[Cd(C_10_H_8_O_4_)(H_2_O)_3_]·2H_2_O	*F*(000) = 792
*M**_r_* = 394.64	*D*_x_ = 1.832 Mg m^−^^3^
Monoclinic, *C*2/*c*	Mo *K*α radiation, λ = 0.71073 Å
Hall symbol: -C 2yc	Cell parameters from 9928 reflections
*a* = 7.6878 (7) Å	θ = 2.7–25.3°
*b* = 8.2295 (8) Å	µ = 1.57 mm^−^^1^
*c* = 22.735 (2) Å	*T* = 173 K
β = 95.752 (1)°	Block, colourless
*V* = 1431.1 (2) Å^3^	0.34 × 0.32 × 0.29 mm
*Z* = 4	

### Data collection

**Table:**

Bruker APEXII CCD diffractometer	1307 independent reflections
Radiation source: fine-focus sealed tube	1296 reflections with *I* > 2σ(*I*)
graphite	*R*_int_ = 0.025
ω–φ scans	θ_max_ = 25.3°, θ_min_ = 1.8°
Absorption correction: multi-scan (*SADABS*; Sheldrick, 1996)	*h* = −9→9
*T*_min_ = 0.621, *T*_max_ = 0.659	*k* = −9→9
11122 measured reflections	*l* = −27→27

### Refinement

**Table:**

Refinement on *F*^2^	Primary atom site location: structure-invariant direct methods
Least-squares matrix: full	Secondary atom site location: difference Fourier map
*R*[*F*^2^ > 2σ(*F*^2^)] = 0.013	Hydrogen site location: inferred from neighbouring sites
*wR*(*F*^2^) = 0.031	H atoms treated by a mixture of independent and constrained refinement
*S* = 1.19	*w* = 1/[σ^2^(*F*_o_^2^) + (0.0098*P*)^2^ + 1.4668*P*] where *P* = (*F*_o_^2^ + 2*F*_c_^2^)/3
1307 reflections	(Δ/σ)_max_ < 0.001
107 parameters	Δρ_max_ = 0.27 e Å^−^^3^
9 restraints	Δρ_min_ = −0.20 e Å^−^^3^

### Special details

**Table:**

Geometry. All e.s.d.'s (except the e.s.d. in the dihedral angle between two l.s. planes) are estimated using the full covariance matrix. The cell e.s.d.'s are taken into account individually in the estimation of e.s.d.'s in distances, angles and torsion angles; correlations between e.s.d.'s in cell parameters are only used when they are defined by crystal symmetry. An approximate (isotropic) treatment of cell e.s.d.'s is used for estimating e.s.d.'s involving l.s. planes.
Refinement. Refinement of *F*^2^ against ALL reflections. The weighted *R*-factor *wR* and goodness of fit *S* are based on *F*^2^, conventional *R*-factors *R* are based on *F*, with *F* set to zero for negative *F*^2^. The threshold expression of *F*^2^ > σ(*F*^2^) is used only for calculating *R*-factors(gt) *etc*. and is not relevant to the choice of reflections for refinement. *R*-factors based on *F*^2^ are statistically about twice as large as those based on *F*, and *R*- factors based on ALL data will be even larger.

### Fractional atomic coordinates and isotropic or equivalent isotropic displacement parameters (Å^2^)

**Table:**

	*x*	*y*	*z*	*U*_iso_*/*U*_eq_	
Cd1	0.0000	0.211515 (18)	0.2500	0.01614 (6)	
O1	0.0000	0.4900 (2)	0.2500	0.0296 (4)	
H1	0.070 (2)	0.552 (2)	0.2346 (8)	0.036*	
O1W	0.21682 (17)	0.51653 (15)	0.09931 (5)	0.0252 (3)	
H1WA	0.199 (3)	0.430 (2)	0.1171 (8)	0.030*	
H1WB	0.307 (2)	0.506 (2)	0.0844 (8)	0.030*	
O2	0.14213 (18)	0.25529 (14)	0.16357 (5)	0.0290 (3)	
O3	0.09896 (14)	0.00183 (13)	0.18665 (5)	0.0208 (2)	
O4	0.27315 (15)	0.20049 (14)	0.30205 (5)	0.0200 (2)	
H4A	0.273 (2)	0.158 (2)	0.3350 (7)	0.024*	
H4B	0.318 (2)	0.2927 (18)	0.3066 (8)	0.024*	
C1	0.1256 (2)	0.0255 (2)	0.04864 (6)	0.0195 (3)	
C2	0.2569 (2)	0.0525 (2)	0.10213 (7)	0.0215 (3)	
H2A	0.3421	0.1368	0.0931	0.026*	
H2B	0.3217	−0.0494	0.1121	0.026*	
C3	0.0777 (2)	−0.1308 (2)	0.03026 (7)	0.0216 (3)	
H3	0.1305	−0.2215	0.0508	0.026*	
C4	−0.0466 (2)	−0.1567 (2)	−0.01772 (7)	0.0217 (3)	
H4	−0.0777	−0.2644	−0.0295	0.026*	
C5	0.1617 (2)	0.10626 (19)	0.15438 (6)	0.0177 (3)	

### Atomic displacement parameters (Å^2^)

**Table:**

	*U*^11^	*U*^22^	*U*^33^	*U*^12^	*U*^13^	*U*^23^
Cd1	0.01982 (9)	0.01563 (9)	0.01342 (9)	0.000	0.00393 (6)	0.000
O1	0.0287 (10)	0.0159 (8)	0.0478 (11)	0.000	0.0214 (8)	0.000
O1W	0.0298 (7)	0.0207 (6)	0.0263 (6)	−0.0005 (5)	0.0085 (5)	0.0015 (5)
O2	0.0469 (8)	0.0178 (6)	0.0247 (6)	0.0008 (5)	0.0153 (6)	−0.0004 (5)
O3	0.0239 (6)	0.0196 (6)	0.0198 (6)	0.0015 (5)	0.0075 (5)	0.0010 (4)
O4	0.0242 (6)	0.0161 (6)	0.0199 (6)	−0.0012 (5)	0.0025 (5)	0.0003 (5)
C1	0.0212 (8)	0.0247 (8)	0.0139 (7)	0.0016 (7)	0.0080 (6)	−0.0005 (6)
C2	0.0205 (8)	0.0273 (9)	0.0174 (8)	0.0022 (7)	0.0055 (6)	−0.0010 (6)
C3	0.0275 (9)	0.0207 (8)	0.0173 (8)	0.0051 (7)	0.0059 (7)	0.0032 (6)
C4	0.0284 (9)	0.0188 (8)	0.0188 (8)	0.0002 (7)	0.0074 (7)	−0.0019 (6)
C5	0.0184 (8)	0.0216 (8)	0.0127 (7)	0.0015 (6)	−0.0012 (6)	−0.0005 (6)

### Geometric parameters (Å, °)

**Table:**

Cd1—O1	2.2917 (17)	O4—H4A	0.827 (14)
Cd1—O4	2.3066 (12)	O4—H4B	0.836 (14)
Cd1—O4^i^	2.3066 (12)	C1—C3	1.391 (2)
Cd1—O2	2.3695 (12)	C1—C4^ii^	1.394 (2)
Cd1—O2^i^	2.3695 (12)	C1—C2	1.517 (2)
Cd1—O3	2.4187 (11)	C2—C5	1.522 (2)
Cd1—O3^i^	2.4187 (11)	C2—H2A	0.9900
O1—H1	0.842 (11)	C2—H2B	0.9900
O1W—H1WA	0.835 (15)	C3—C4	1.392 (2)
O1W—H1WB	0.810 (15)	C3—H3	0.9500
O2—C5	1.256 (2)	C4—C1^ii^	1.394 (2)
O3—C5	1.2571 (19)	C4—H4	0.9500
			
O1—Cd1—O4	92.25 (3)	Cd1—O4—H4A	113.4 (13)
O1—Cd1—O4^i^	92.25 (3)	Cd1—O4—H4B	111.8 (13)
O4—Cd1—O4^i^	175.49 (6)	H4A—O4—H4B	108.1 (18)
O1—Cd1—O2	81.25 (3)	C3—C1—C4^ii^	118.40 (15)
O4—Cd1—O2	87.69 (4)	C3—C1—C2	120.78 (15)
O4^i^—Cd1—O2	93.00 (4)	C4^ii^—C1—C2	120.79 (15)
O1—Cd1—O2^i^	81.25 (3)	C1—C2—C5	109.59 (13)
O4—Cd1—O2^i^	93.00 (4)	C1—C2—H2A	109.8
O4^i^—Cd1—O2^i^	87.69 (4)	C5—C2—H2A	109.8
O2—Cd1—O2^i^	162.51 (6)	C1—C2—H2B	109.8
O1—Cd1—O3	135.51 (3)	C5—C2—H2B	109.8
O4—Cd1—O3	87.30 (4)	H2A—C2—H2B	108.2
O4^i^—Cd1—O3	89.48 (4)	C1—C3—C4	121.14 (15)
O2—Cd1—O3	54.27 (4)	C1—C3—H3	119.4
O2^i^—Cd1—O3	143.22 (4)	C4—C3—H3	119.4
O1—Cd1—O3^i^	135.51 (3)	C3—C4—C1^ii^	120.47 (16)
O4—Cd1—O3^i^	89.48 (4)	C3—C4—H4	119.8
O4^i^—Cd1—O3^i^	87.30 (4)	C1^ii^—C4—H4	119.8
O2—Cd1—O3^i^	143.22 (4)	O2—C5—O3	120.75 (14)
O2^i^—Cd1—O3^i^	54.27 (4)	O2—C5—C2	119.26 (14)
O3—Cd1—O3^i^	88.97 (5)	O3—C5—C2	119.96 (14)
Cd1—O1—H1	127.1 (12)	O2—C5—Cd1	59.31 (8)
H1WA—O1W—H1WB	107.7 (19)	O3—C5—Cd1	61.56 (8)
C5—O2—Cd1	93.58 (9)	C2—C5—Cd1	177.81 (11)
C5—O3—Cd1	91.25 (9)		
			
O1—Cd1—O2—C5	178.70 (10)	Cd1—O2—C5—O3	4.15 (16)
O4—Cd1—O2—C5	86.06 (10)	Cd1—O2—C5—C2	−178.08 (12)
O4^i^—Cd1—O2—C5	−89.48 (10)	Cd1—O3—C5—O2	−4.05 (15)
O2^i^—Cd1—O2—C5	178.70 (10)	Cd1—O3—C5—C2	178.19 (12)
O3—Cd1—O2—C5	−2.28 (9)	C1—C2—C5—O2	−92.22 (18)
O3^i^—Cd1—O2—C5	0.02 (14)	C1—C2—C5—O3	85.57 (18)
O1—Cd1—O3—C5	3.65 (11)	O1—Cd1—C5—O2	−1.36 (10)
O4—Cd1—O3—C5	−86.82 (9)	O4—Cd1—C5—O2	−92.46 (10)
O4^i^—Cd1—O3—C5	96.34 (9)	O4^i^—Cd1—C5—O2	91.83 (10)
O2—Cd1—O3—C5	2.27 (9)	O3—Cd1—C5—O2	175.95 (15)
O2^i^—Cd1—O3—C5	−178.22 (9)	O3^i^—Cd1—C5—O2	−179.99 (9)
O3^i^—Cd1—O3—C5	−176.35 (11)	C5^i^—Cd1—C5—O2	178.64 (10)
C5^i^—Cd1—O3—C5	−178.21 (5)	O1—Cd1—C5—O3	−177.31 (8)
C3—C1—C2—C5	−104.15 (17)	O4—Cd1—C5—O3	91.60 (9)
C4^ii^—C1—C2—C5	74.03 (18)	O4^i^—Cd1—C5—O3	−84.12 (9)
C4^ii^—C1—C3—C4	−0.2 (3)	O2—Cd1—C5—O3	−175.95 (15)
C2—C1—C3—C4	178.06 (14)	O3^i^—Cd1—C5—O3	4.07 (12)
C1—C3—C4—C1^ii^	0.2 (3)	C5^i^—Cd1—C5—O3	2.70 (8)

Symmetry codes: (i) −*x*, *y*, −*z*+1/2; (ii) −*x*, −*y*, −*z*.

### Hydrogen-bond geometry (Å, °)

**Table:**

*D*—H···*A*	*D*—H	H···*A*	*D*···*A*	*D*—H···*A*
O1—H1···O4^iii^	0.84 (1)	1.96 (1)	2.8029 (15)	178 (2)
O1W—H1WA···O2	0.84 (2)	1.86 (2)	2.6934 (17)	172.(2)
O4—H4A···O1W^iv^	0.83 (1)	1.89 (2)	2.7004 (17)	167.(2)
O4—H4B···O3^iii^	0.84 (1)	1.84 (2)	2.6706 (16)	175 (2)

Symmetry codes: (iii) −*x*+1/2, *y*+1/2, −*z*+1/2; (iv) −*x*+1/2, *y*−1/2, −*z*+1/2.
